# Risk factor score for the prediction of central compartment lymph node metastasis in papillary thyroid carcinoma and its clinical significance

**DOI:** 10.3389/fsurg.2022.914696

**Published:** 2022-11-07

**Authors:** Xiaojia Pan, Qinghuai Li

**Affiliations:** ^1^ Department of General Surgery, Xingtai People Hospital, Xingtai, China; ^2^ Department of Thyroid and Breast Surgery, the Second Hospital of Hebei Medical University, Shijiazhuang, China

**Keywords:** thyroid carcinoma, central compartment lymph node, lymph node metastasis, risk factor score, surgery

## Abstract

**Objective:**

To establish the criteria for a risk factor score (RFS) for predicting the probability of central compartment lymph node metastasis (LNM) in papillary thyroid carcinoma (PTC) and to explore the clinical significance of the RFS.

**Methods:**

The data of 412 patients with PTC who underwent surgical resection between May 2013 and July 2016 were retrospectively analysed and divided into two groups: a central LNM group and a non-central LNM group. In each group, the frequency of six risk factors was documented: sex, age, tumour size, extracapsular spread (ECS), tumour multifocality, and tumour location. The maximum likelihood method of discriminant analysis was adopted to calculate patient scores for the six risk indicators. In addition, the data of 104 patients with PTC admitted between July 2016 and December 2016 were prospectively analysed using this method and these six risk factors. A higher score represented one certain possibility that was the more appropriate for one patient.

**Results:**

In the retrospective group, the result was as follows: 129 patients with positive (+) lymph nodes in the central compartment and 168 patients with negative (−) lymph nodes in the central compartment, which was in line with the actual results. In the prospective group, there were 28 patients with positive lymph nodes in the central compartment and 48 patients with negative lymph nodes in the central compartment. The coincidence rates using the RFS were 71.9% for the retrospective group and 73.1% for the prospective group.

**Conclusion:**

By simple and quantitative analyses of the presence of central LNM, the RFS is of great significance when choosing surgical approaches and postoperative individual-based treatment plans, as well as when determining the prognosis of central compartment LNM in patients with PTC.

## Introduction

Thyroid carcinoma is the most common endocrine malignancy and a common head and neck malignancy. Papillary thyroid carcinoma (PTC), specifically, is a malignant tumour derived from the thyroid follicular epithelial cells that accounts for about 85% to 90% of thyroid cancers (1). Although PTC has a low degree of malignancy and a good prognosis, it is prone to either cervical lymph node metastases (LNM) or distant metastases at the time of diagnosis, and the central lymph node metastasis rate can be as high as 80% (2–4). The most fundamental and effective method of treating PTC primary tumours and lymph node metastases is surgery. In terms of patients with thyroid cancer who have suspected or confirmed central compartment LNM, therapeutic central lymph node dissection (CLND) is the mainstay of treatment, but one controversial issue is whether routine prophylactic dissection should be performed in patients without evident cervical LNM (5). If CLND is not performed in carcinoma cases, residual tumours are induced. When reoperation is required, it will inevitably lead to an increased risk of surgical complications due to the formation of surgical scars and the disruption of the anatomical hierarchy. Currently, ultrasonography (US) is the optimal imaging method in thyroid and regional lymph node examinations, and it has a high sensitivity to benign and malignant thyroid nodules and high accuracy for identifying them (6). However, due to the anatomic location of the central lymph nodes and for certain technical reasons, the diagnostic sensitivity is low (merely 31.3%) (7–12). For this paper, data of patients with PTC were retrospectively analysed using the maximum likelihood method to obtain the scores of factors associated with central LNM. The factors are sex, age, tumour diameter, extracapsular spread (ECS), multifocality, and tumour location. Sex: In thyroid cancer, female patients are more of a concern because of a higher incidence rate. However, several studies suggest that the rate of cervical LNM in men is higher than in women (3, 13, 14). Age: This has been an important factor in various staging systems for differentiated PTC (13, 15, 16). Tumour diameter and ECS: Both have always been considered to be important factors in the progression of PTC and are important criteria for evaluating treatment options and surgical scope, and larger tumour diameters are associated with higher cervical LNM rates and T staging (17). Multifocality: PTC often leads to intraglandular metastasis, and multifocality is one of its prominent features. According to literature reports, multifocal carcinoma accounts for 20.3%–33.5% of cases of PTC (14, 16, 18). Tumour location: In some digestive tract tumours, such as gastric cancer and colon cancer, LNM is closely related to the lymphatic flow path in the region where the tumour is located (19, 20). Therefore, we speculate that PTC LNM is related to the lymphatic flow path of the thyroid region where the tumour is located. The criteria for the risk factor score (RFS) were used to prospectively evaluate another group of 104 patients diagnosed with PTC, as well as the clinical significance of the RFS.

## Materials and methods

### General information

#### Retrospective data

We enrolled 412 patients admitted to our hospital between May 2013 and July 2016 who were diagnosed by postoperative paraffin pathology as having PTC. There were 90 males and 322 females in the study, with a sex ratio of 1:3.6 and a mean age of 44.5 (range 14–81 years old). Tumour diameters ranged from 0.2 to 3.8 cm, with an average diameter of 1.22 cm. A total of 81 patients had ECS (19.6%) and 331 patients did not have ECS. In addition, 169 patients (41%) had papillary thyroid microcarcinoma (PTMC), and 197 patients had central LNM, with a metastasis rate of 47.9%.

#### Prospective data

A total of 104 patients admitted to our department between July 2016 and December 2016 were diagnosed by postoperative paraffin pathology as having PTC. There were 24 males and 80 females with a mean age of 46.6 (range 20–73 years old). The mean tumour diameter was 1.19 cm (range 0.3–3.8 cm). The number of patients with and without ECS was 9 (8.7%) and 95 (91.3%), respectively. A total of 48 patients had PTMC (46%) and 41 patients had central LNM (39.4%).

### Inclusion and exclusion criteria

Inclusion criteria were as follows: (1) neck surgery for the first time; (2) ipsilateral CLND performed; and (3) complete medical records, including a B-ultrasound examination and postoperative paraffin pathology. Patients with the following diagnoses were excluded: (1) metastatic thyroid cancer; (2) thyroid cancer combined with other types of undifferentiated carcinoma; and (3) multiple foci in two or more different parts (the upper, middle, and lower poles, as well as the isthmus).

### Clinical observation criteria

Six factors predictive of central LNM were described: (1) sex: both male and female; (2) age: <45 years old and ≥45 years old; and (3) tumour diameter: <0.7 cm, 0.7–1.0 cm, 1.0–1.5 cm, 1.5–2.0 cm, and ≥2.0 cm; (4) ECS: presence or absence; (5) multifocality: presence or absence; and (6) tumour location: the upper (upper 1/3 of the gland), the middle (middle 1/3 of the gland) and the lower pole (lower 1/3 of the gland), as well as the isthmus. A frequency table of count data was constructed in accordance with these six indicators based on data collected from the 412 patients in the retrospective study (Table 1).

**Table 1:** Frequency of six indicators for central LNM in the retrospective group (No. of cases).

Risk factor		Central compartment lymph node	
		+	−
Sex	Female	144	178
	Male	53	37
Age	<45 years old	111	95
	≥45 years old	86	120
Tumour diameter	<0.7 cm	18	74
	0.7–1.0 cm	37	55
	1.0–1.5 cm	53	62
	1.5–2.0 cm	35	16
	≥2.0 cm	54	8
ECS	Presence	61	20
	Absence	136	195
Multifocality	Presence	13	9
	Absence	184	206
Tumour location	Upper pole	26	48
	Middle pole	106	111
	Lower pole	57	45
	Isthmus	8	11

### Treatment

Ultrasonography was performed by two sonographers with more than 5 years of experience. The central lymph node status was recorded. The extent of thyroidectomy was determined according to the clinical examination results or preoperative biopsy results. At least unilateral gland lobe plus isthmus resection and total thyroidectomy or near-total thyroidectomy should be performed if any of the following conditions are met: distant metastasis, the primary tumour is larger than 4 cm, the primary tumour invades the surrounding tissue, or a multifocal tumour or cervical LNM can be seen with the naked eye. The histology of frozen sections further helps surgeons determine the extent of surgery required.

A total of 412 patients in the retrospective group underwent standardised surgical treatment: thyroid lobectomy, isthmus resection, and ipsilateral CLND (*n* = 168); total thyroidectomy and ipsilateral CLND (*n* = 190); and total thyroidectomy plus bilateral CLND (*n* = 54). In the prospective study of 104 patients, the above operation methods were used in 46 cases, 28 cases, and 30 cases, respectively. All patients who were given oral levothyroxine sodium tablets underwent thyroid-stimulating hormone (TSH) inhibition treatment. One month later, thyroid function tests were performed, and the dosage of Euthyrox was tailored according to the TSH level. During this period, thyroid function tests were reperformed every 1 and a half months. B-ultrasound examinations of the thyroid and neck lymph nodes were conducted half a year after surgery and every half a year thereafter, as well as other tests such as physical examinations, thyroid function tests, ECGs, and chest x-rays. All patients were advised to see a doctor if necessary.

### Postoperative follow-up

All patients were followed up for one to 4 years, with an average of 28.3 months, apart from three who were lost to the study due to changes in telephone numbers. Some patients had postoperative transient hypocalcaemia and hoarseness. Nonetheless, all of them recovered within 6 months of surgery. No other serious complications arose.

### Statistical method

As given in Table 1, the frequency (P) of the six indicators for central LNM was calculated using the formula (X_kj_/Y_g_), in which Y represents the type of the central lymph node: g = 1 indicates positive (+), and g = 2 indicates negative (−). X_kj_ (k = 1,2,3,4,5,6; j = 1,2) is the jth classification of the kth indicator. The maximum likelihood method of discriminant analysis for qualitative data was used to estimate the probability of type g central lymph node in X_kj_ patients: P_g _= P (X_1j_/Y_g_)×P (X_2j_/Y_g_)…×P (X_6j_/Y_g_).

Take the logarithm of both sides: lgP_g _= lgP (X_1j_/Y_g_) + lgP (X_2j/_Y_g_)…+lgP (X_6j_/Y_g_). The score of each indicator was derived from [lgP (X_kj_/Y_g_)+1]×10 (Table 2).

**Table 2:** Scores of all indicators through the discriminant analysis.

Risk factor		Central compartment lymph node	
		+	−
Sex	Female	8.6	9.2
	Male	4.3	2.4
Age	<45 years old	7.5	6.5
	≥45 years old	6.4	7.5
Tumour diameter	<0.7 cm	−0.4	5.4
	0.7–1.0 cm	2.7	4.1
	1.0–1.5 cm	4.3	4.6
	1.5–2.0 cm	2.5	−1.3
	≥2.0 cm	4.4	−4.3
ECS	Presence	4.9	−0.3
	Absence	8.4	9.6
Multifocality	Presence	−1.8	−3.8
	Absence	9.7	9.8
Tumour location	Upper pole	1.2	3.5
	Middle pole	7.3	7.1
	Lower pole	4.6	3.2
	Isthmus	−3.9	−2.9

The scoring formula was as follows: Sg = {[lgP (X_1j_/Y_g_)+1] + [lgP (X_2j_/Y_g_)+1]…+[lgP (X_6j_/Y_g_)+1]}×10.

### Statistical analysis

In this study, the above formula was used for discriminant analysis of the data from the 104 patients in the prospective group and for back substitution of the data from the 412 patients in the retrospective group. The Wilcoxon signed-rank test of two independent samples was performed for comparison between the retrospective and the prospective groups. The significance for all variables was set at α = 0.05. SPSS 21.0 software was used for statistical analyses.

## Results

**Figure 1:**
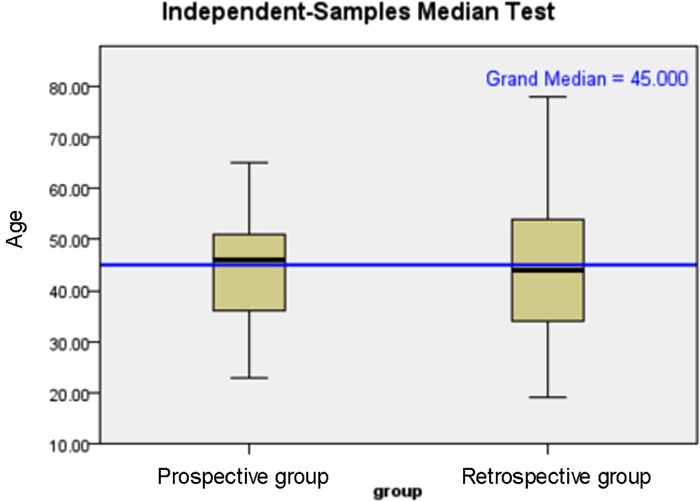
Age distribution of patients in prospective and prospective groups.

**Figure 2:**
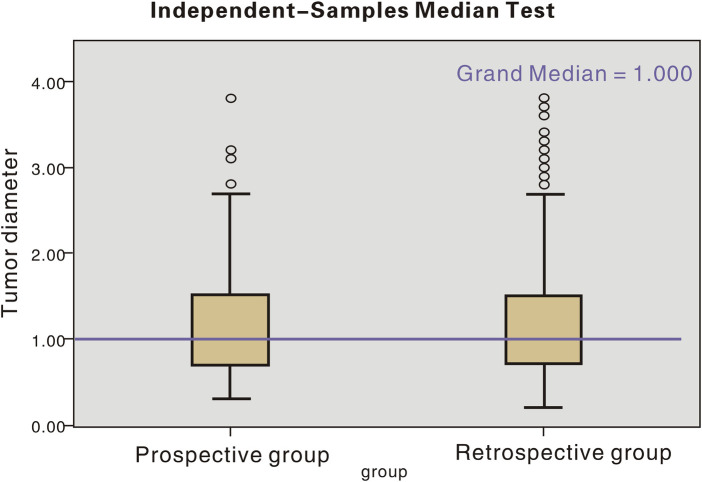
Tumour diameter distribution in patients in retrospective and prospective groups.

The age distribution and tumour diameter distribution of the two groups are shown in Figures 1, 2, and the score of each factor related to central LNM is given in Table 2. In the retrospective group, the discriminant analysis result was as follows: 129 patients had positive (+) lymph nodes in the central compartment and 168 patients had negative (−) lymph nodes in the central compartment, which was in line with actual results. The coincidence rates were 65.5% and 78.1%, respectively, and the overall coincidence rate was 71.8% (Table 3). However, in the prospective group, 28 patients had positive lymph nodes in the central compartment and 48 patients had negative lymph nodes in the central compartment. The coincidence rates were 68.2% and 76.2% respectively, with an overall coincidence rate of 72.2% (Tables 3, 4).

**Table 3:** Evaluation of discrimination in the retrospective group (No. of cases).

Actual result	Discriminate result		Total	Coincidence rate (%)
	+	−		
+	129	68	197	65.5
−	47	168	215	78.1
Mean	—	—	—	71.8

**Table 4:** Evaluation of discrimination in the prospective group (No. of cases).

Actual result	Discriminate result		Total	Coincidence rate (%)
	+	−		
+	28	13	41	68.2
−	15	48	63	76.2
Mean	—	—	—	72.2

## Discussion

PTC is a common disease in clinical settings. A B-ultrasound examination is currently an effective method of distinguishing between benign and malignant thyroid nodules. Dan et al. (21) reported that the diagnosis rate of PTC was as high as 87.64% by high-frequency colour ultrasound, which was, however, not sensitive in the detection of central LNM [approximately 31.3% (7–12)], much lower than the detection rate of lateral neck metastasis (93.8%) (22). This may be related to the anatomical location of the central lymph nodes (adjacent to thyroid and gas-containing trachea) and available technology.

Central LNM is associated with a number of factors. The relevant factors involved in this study were as follows: (1) Sex: Male patients are more likely to experience LNM than female patients (23). Of the 516 patients enrolled, 66 of 114 males developed central LNM and 172 of 402 females. The metastasis rates were 57.9% and 42.8%, respectively. (2) Age: The cut-off age of 45 is widely used as a clinical marker in the American Joint Committee on Cancer TNM staging system guideline (2012 Chinese version) (24). In Western literature, an age of >45 was referred to as a risk factor for thyroid cancer. It is noteworthy that Ito et al. (25) demonstrated an association between a younger age and a higher rate of LNM. In addition, patients 20 years old or younger had a metastasis rate of up to 50%. (3) Tumour diameter: According to National Comprehensive Cancer Network guidelines, a tumour diameter of >1 cm is a risk factor for cervical LNM (26), and patients with PTC with tumour diameters of >2 cm had a higher LNM rate in the central region and the lateral neck than those with tumour diameters of ≤2 cm (27). 0.5 or 0.6 cm has been recognised as the cut-off value of central LNM in PTC. In our previous studies (28), a tumour diameter of ≥0.7 cm was of statistical significance in the prediction of central LNM in PTMC. (4) ECS: Based on studies by Radowsky et al. (29), ECS is an important factor affecting the prognosis of PTC. Extrathyroidal extension contributed to increased invasion associated with a weakened inhibitory effect of extracellular matrix on LNM in PTC and capsular invasion of the dense network in the thyroid (30). (5) In terms of multifocality, Kuo et al. (31) found that multifocal primary PTMC had a significantly increased metastasis rate compared with unifocal PTMC, consistent with the findings in our previous studies. (6) Tumour location: Zhang et al. (14) reported that a tumour located in the upper pole of the gland was associated with an increased risk of LNM in the lateral neck but a decreased risk of central LNM. Wang et al. (32) found that patients with tumours located in the middle and lower poles are more likely to experience central LNM. Based on the data in Table 1, the rates of central LNM in tumours located in the upper, middle, and lower poles and the isthmus were 35.1%, 48.8%, 55.9%, and 42.1%, respectively.

There has been no international consensus regarding prophylactic CLND in patients with cN0 PTC. In this study, the frequency of each relevant factor was measured based on retrospective data. Likelihood function discriminant analysis was used in the prospective group, and samples were subject to back substitution to evaluate the method's effectiveness. Given unchanged discriminant rules in the likelihood function after logarithm transformation, scoring criteria were developed and applied as follows: With respect to a 35-year-old female PTC patient with a tumour diameter of 1.6 cm, single lesions and no extracapsular spread, and with the tumour located in the lower pole of the lobes, the score was Spositive = 41.3, Snegative = 37, and Spositive > Snegative, indicating the presence of central LNM.

In this study, statistical methods were used for specific and quantitative discriminant analysis of central LNM and benign lesions in patients with PTC. The accuracy (approximately 72%) was superior to the current discriminant analysis of B-ultrasound measurements, which is of clinical significance. Several studies have reported that central LNM is also associated with the coexistence of Hashimoto's thyroiditis and higher TSH levels in patients.

The present study has several limitations. First, the above factors have not been included, and a multicentre study has not been conducted, leading to a certain selection bias, which should ideally be avoided. Further study is, therefore, needed to standardise the scoring criteria. Second, the current follow-up timeframe has not been long enough compared with disease development. We will continue to follow up on these patients. Another limitation of the current study is the small number of patients, and further studies with larger samples are needed. Furthermore, the time of the study has a certain bias, and the results need to be further verified in a subsequent study at a different time. Last, we have not analysed the relationship between Hashimoto's thyroiditis and PTC, and we will add this feature as a part of the risk factor score.

## Data Availability

The original contributions presented in the study are included in the article/Supplementary Material, and further inquiries can be directed to the corresponding author/s.
